# Genome-wide analysis of CHYR gene family and *BnA03.CHYR.1* functional verification under salt stress in *Brassica napus* L.

**DOI:** 10.1186/s12870-025-06343-x

**Published:** 2025-03-20

**Authors:** Yanli Guo, Qingxiao Ren, Manman Song, Xiangxiang Zhang, Heping Wan, Fei Liu

**Affiliations:** 1 College of Horticulture and Landscape Architecture, Tianjin Agricultural University, Tianjin, 300392 China; 2 State Key Laboratory of Crop Stress Adaption and Improvement, School of Life Sciences, Henan University, Kaifeng, Henan 475004 China; 3 Jianghan University/Hubei Engineering Research Center for Conservation Development and Utilization of Characteristic Biological Resources in Hanjiang River Basin, Wuhan, 430056 China; 4 College of Life Sciences, Nankai University, Tianjin, 300071 China; 5 Oil Crops Research Institute of the Chinese Academy of Agricultural Sciences, Wuhan, 430062 China

**Keywords:** *Brassica napus*, CHYR, Abiotic stress, Ubiquitin E3 ligase, *BnA03.CHYR.1*

## Abstract

*Brassica napus*, an allotetraploid used as an oilseed crop, vegetable, or feed crop, possesses significant economic and medicinal value. Although the CHYR gene family has been functionally characterised in various aspects of plant growth, development, and stress responses, its systematic investigation in *B. napus* is lacking. In contrast to the seven CHYR genes (*AtCHYR1-AtCHYR7*) identified in *Arabidopsis thaliana*, nine CHYR orthologues were detected in *B. rapa* and *B. oleracea*, while 24 were found in *B. napus*. This discrepancy is consistent with the established triplication events that occurred during the Brassicaceae family evolution. Phylogenetic analysis indicated that the 24 CHYRs identified in *B. napus* could be categorised into three distinct groups. Among these, 24 BnCHYRs contained conserved domains, including the CHY-zinc finger, C3H2C3-type RING finger and zinc ribbon domains. Group III members featured an additional one to three hemerythrin domains in their N-terminal regions. Each *BnCHYR group* shared similar patterns in the distribution of conserved domains. Our results revealed that the selected eight *BnCHYRs* were up-regulated following heat treatment, exhibiting varying expression patterns in response to salt, cold, and drought stress during the seedling stage. Expression analysis revealed that several *BnCHYRs* were significantly induced by one or more abiotic stressors. *BnA03.CHYR.1* was significantly induced by salt and heat stress and repressed by polyethylene glycol treatment. *BnA03.CHYR.1* was localised in the nucleus and cytoplasm, and its overexpression in *A. thaliana* enhanced tolerance to salt stress. Our results provide a comprehensive analysis of the *CHYR* family in *B. napus*, elucidating the biological role of *BnA03.CHYR.1* in adaptive responses of plants to salt stress.

**Supplementary Information:**

The online version contains supplementary material available at 10.1186/s12870-025-06343-x.

## Introduction

*Brassica napus* (*B. napus;* AACC, 2 *n* = 38) is an essential oilseed and vegetable crop [1], with many agronomic advantages, such as rapid growth, high biomass productivity, and strong adaptability to diverse environmental conditions [2–4]. Plants usually encounter various abiotic and biotic stresses during their life cycle, such as salinity, dehydration, extreme temperatures, and pathogen infection [5]. As sessile organisms, plants have evolved sophisticated biochemical and physiological responses to contend with various environmental challenges such as drought [6, 7], high salinity [8], wounding, and temperature extremes [9–11]. The production and quality of *B. napus* are significantly affected by adverse environmental conditions [12, 13]. Therefore, it is critical to improve stress tolerance in *B. napus* by identifying and using genes involved in the stress response.

According to conserved motif and phylogenetic relationship analyses, CHYR (CHY zinc-finger and RING finger) proteins are classified into three groups: I, II, and III [14]. CHYR belongs to the RING-type E3 ubiquitin ligase family and is an essential stress-responsive protein that responds to abiotic stress in plants [14, 15]. The CHYR, containing CHY zinc finger, C3H2C3-type ring finger, and rubredoxin-type fold domain, was named for the conserved “CxHY” motif at the domain starting position [15]. According to the conserved motifs and phylogenetic relationship analyses, CHYR contains 12 cysteines and histidines, forming a special zinc finger that participates in protein interactions, ubiquitination, and zinc ion binding [15–18]. The C3H2C3-type RING finger domain, also referred to as the RING-H2 finger, exists at the CHYR C-terminus, can bind to two zinc atoms, and may be involved in protein–protein interactions [16, 19]. In addition, Group III members, also known as BRUTUS/BRUTUS-like (BTS/BTSL) or hemerythrin motif-containing RING and zinc- finger (HRZ) proteins, contain N-terminal hemerythrin domains and play an essential role in regulating iron homeostasis [20–23].

RING E3 proteins play critical roles in abiotic stress responses via protein ubiquitination and degradation [24, 25]. CHYR proteins belong to the RING-type E3 ubiquitin protein ligase family and perform vital functions in plant growth, development, and various stress responses [15, 20, 26]. *AtCHYR1* positively regulates stomatal closure and improves drought tolerance via SnRK2.6-mediated phosphorylation [15]. Similarly, AtCHYR1 ubiquitinates phosphorylated WRKY DNA binding protein 70, marking it for degradation to modulate the balance between immunity and growth [27]. *PeCHYR1* isolated from *Populus euphratica*, enhanced drought tolerance via abscisic acid (ABA)-induced stomatal closure caused by hydrogen peroxide production in transgenic poplar plants [28]. *SlCHYR1*, isolated from tomato (*Solanum lycopersicum* L.), encodes a protein that promotes fruit ripening by reprogramming ABA and ethylene signalling [29]. However, the *AtCHYR1* homologous gene rice (*Oryza sativa*) *OsRZF34* enhances stomatal opening, leaf cooling, and ABA insensitivity [30]. The RING-type ubiquitin E3 ligase MYB30-interacting E3 ligase11 (MIEL1) participates in the hypersensitivity response by mediating MYB30 degradation, which is a crucial activator of this response [26]. MIEL1 negatively regulates cuticular wax biosynthesis in *A. thaliana* stems [31] and localises to peroxisomes to promote seedling oleosin degradation and lipid droplet mobilisation [32]. *A. thaliana* MIEL1 directly mediates ABI5 proteasomal degradation and inhibits its activity via the release of its target protein, MYB30, ensuring precise ABA signalling during seed germination and seedling establishment [33]. *A. thaliana* BTS plays a crucial role in the drought stress response by facilitating the degradation of transcription factor vascular plant one-zinc finger 1/2 protein [34]. CHYR proteins with two to three additional hemerythrin domains (BST/BTSL/HRZ) also play an essential role in regulating iron responses in *A. thalianas* and rice [20, 21, 23, 35–39]. *AtCHYR2* is a cytoplasmic RING ubiquitin E3 ligase that plays a vital role in the glucose response [40].

Despite extensive studies on the CHYR family in many species, such as maize, *A. thaliana*, rice, bread wheat, soybean, and *Sophora alopecuroides* [14, 20, 30, 40–43], the genome-wide identification of CHYR in rapeseed has not been well characterised. To explore the structural diversity and evolution of BnCHYRs, we identified the CHYR family comprising 24 genes in *B. napus* and analysed their phylogenetic relationships, gene structures, conserved motifs, and *cis*-acting elements in the promoter region. In the present study, we identified 24 BnCHYRs in the genomes of U-triangle species and comprehensively analysed the features of their encoded proteins. Additionally, we investigated *BnCHYR* expression profiles by focusing on their responses to several abiotic stresses.

## Materials and methods

### Plant materials and treatments

The *B. napus* variety “Westar” was used as the experimental material, and *A. thaliana* (*A. thaliana* ecotype Columbia [*Col*−0]) was used for genetic transformation in this study. Seeds of “Westar” and “*col*−0” were obtained from the Hubei Engineering Research Center for Conservation, Development and Utilization of Characteristic Biological Resources in Hanjiang River Basin of Jianghan University. Plants of these two species were grown in an illumination incubator with a constant temperature and light cycle (16 h light/8 h dark; 22 ± 2℃; light intensity approximately 12,000 lx; humidity 80%). *B. napus* seeds germinated on moist gauze for three days were transferred to 1/2-strength Hoagland’s solution and allowed to grow for approximately 21 days. To examine *BnCHYR* expression pattern in response to various stress treatments, we exposed five independent biological replicates with similar growth potential (24℃; 16 h of light /8 h dark; light intensity 12,000 lx, relative humidity 80%) to 0.15 M NaCl solution (for salinity stress), a temperature of 4℃ (for cold stress on water-soaked filter paper), 15% polyethylene glycol-6000 solution (for drought stress), and a temperature of 40℃ (for heat stress); untreated seedlings served as the control. Five independent biological replicates were performed for each treatment. The above treated seedling samples were collected at 0, 3, 6, 12, and 24 h. Furthermore, all experimental seedling tissue samples were frozen in liquid nitrogen immediately and stored at −80℃ for RNA extraction by quantitative real-time polymerase chain reaction (RT-qPCR) experiments. To study the effect of NaCl on germination and seedling, we planted seeds on 1/2MS medium supplemented with 1% sucrose and varying NaCl concentrations in a growth chamber at 22℃ under a 16 h light/8 h dark photoperiod with 60% relative humidity.

### Identification of CHYR gene family members from *B. napus, B. rapa, and B. oleracea*

To identify CHYRs, we retrieved the sequences of seven AtCHYRs from the *A*. *thaliana* genome (http://www.arabidopsis.org/) and subsequently used them to identify CHYR genes in the genomes of *B. napus* (BnPIR, http://cbi.hzau.edu.cn/bnapus) *B. rapa* (Brara_Chiifu_V3.0, http://brassicadb.cn/), and *B. oleracea* (Brara_Chiifu_V3.0, Braol_JZS_V2.0; Footnote 3) via reciprocal BLAST using the BLASTP program [44]. Default parameters with E-values < 1E-10 were set in the BLASTP search.

### Phylogenetic analysis of the *CHYR* gene

The CHYR protein sequences of *B. napus*, *B. rapa*, *B. oleracea*, and *A. thaliana* were merged, and multiple sequence alignments were performed using ClustalW [45]. The phylogenetic tree was constructed by the neighbor-joining method with 1000 bootstrap replicates using MEGA11 software [46]. The interactive Tree of Life (iTOL, https://itol.embl.de) was used to visualize the evolutionary tree [47].

### Chromosomal mapping, duplicated type, and collinear block analysis

Chromosomal position information for *BnCHYRs* was extracted from generic feature format (GFF) files downloaded from the *B. napus* genome website (see footnote 2). The positions of *BnCHYRs* were indicated on the corresponding chromosomes using TBtools software [48]. Multiple Collinearity Scan Toolkit (MCscanX; https://github.com/wyp1125/MCScanx) was used to identify gene duplication types and collinearity relationships [49]. Gene duplication analysis was performed using the MCScan X program with default parameters, and the locationand the collinearity relationships of these gene pairs were displayed using the Circos software [50].

### Structure, conserved motifs and physio-chemical properties of BnCHYR proteins

The physico-chemical propertied including molecular weight, theoretical PI, instability index, aliphatic index, and grand averge of hydropathy (GRAVY) of BnCHYR proteins were evaluated using the ExPaSy’s ProParam tool (http://web.expasy.org/protparam/). The subcellular localization of BnCHYR proteins was predicted by the Cellular Localization of Proteins (Cell-PLoc; https://www.csbio.sjtu.edu.cn/bioinf/Cell-PLoc-2/ ). Gene structures including UTRs, introns, and exons, were shown using TBtools software (V1.068; https://githgu.com/CJ-Chen/TBtools) [48]. The conserved motifs of BnCHYR protein sequences were identified using the MEME program (https://meme-suite.org/meme/db/motifs) with default parameters [51].

### Cis-acting prediction in the promoter of BnCHYR genes

*BnCHYR* promoter information was extracted from *B. napus* GFF files, and promoter sequences were isolated using the seqtk software [52]. Furthermore, 2000 bp upstream sequences of the coding region were obtained and submitted to PlantCare (http://bioinformatics.psb.ugent.be/webtools/plantcare/html/) for *cis*-acting element analysis [53], and the results were sorted and displayed using TBtools [48].

### Transformation of *A. thaliana*

The CaMV 35S promoter has been shown to function efficiently in a wide range of plants, including *Physcomitrella* [54]. To generate *BnA03.CHYR.1* overexpression lines (OEs), *A. thaliana Columbia* ecotype (*Col*−0) was transformed with *A. tumefaciens* GV3103 harboring the pBinGlyRed3-35S-BnA03.CHYR.1 plasmid using the floral dipping method [55]. T_1_ transgenic plants were selected from T_0_ plants based on kanamycin resistance and further confirmed for T-DNA integration using PCR. T_2_ transgenic plants with kanamycin resistance segregation ratios of 3:1 were selected, and *BnA03.CHYR.1* expression levels were subsequently analysed using RT-qPCR. Three lines with high *BnA03.CHYR.1* expression levels were continually screened until homozygous lines were obtained and used for subsequent functional analysis. The primers used for PCR and RT-qPCR are listed in Supplemental Table S1.

### RNA extraction and RT-qPCR

Total RNA was extracted from oilseed rape seedlings using the RNAprep plant kit (TIANGEN, DP441, China). Two micrograms of total RNA was used for cDNA synthesis using the RevertAid First Strand cDNA kit (Fermentas, #K1622, USA).

RT-qPCR analysis was performed using the CFX96 Real-Time system (Bio-Rad, USA) using the SYBR Green Realtime PCR Master Mix (TOYOBO, OPK-201, Japan). BnACTIN7 was used as a control to normalize expression levels according to the 2^–ΔΔCT^ method of analysis [56]. The RT-qPCR primers are listed in Supplemental Table S1.

### Subcellular localisation analysis

The *BnA03.CHYR.1* coding sequence (CDS) without a stop codon was cloned and inserted into the pCAMBIA1300-35S-sGFP vector. After confirmation through sequencing, pCAMBIA1300-35S-BnA03.CHYR.1::GFP and pCAMBIA1300-35S-sGFP (positive control) plasmids were introduced into *Agrobacterium tumefaciens* GV3101. *Agrobacterium tumefaciens* transformed with the target vector and nuclear localization marker (NLS-RFP) were mixed in equal proportions and infiltrated into *Nicotiana benthamiana* leaves for transient expression. After growing for one and two days in the dark and light, respectively, tobacco leaf was observed using a confocal microscope (Zeiss, LSM710; Jena, Germany). The primers used for gene cloning and vector construction are listed in Supplemental Table S1.

## Results

### Identification and characterisation of BnCHYRs

In this study, we identified 24 BnCHYRs in *B. napus* using the known seven *A. thaliana* CHYR peptide sequences as queries and performed BLASTP searches in the *B. napus* genome database (BnPIR, http://cbi.hzau.edu.cn/bnapus). Detailed characteristics of the 24 BnCHYRs are presented in Table 1. 11 genes were positioned in the An-sub-genome and 12 were positioned in the Cn-sub-genome (Table 1). To confirm BnCHYR integrity, we analysed the retrieved sequences in the *B. napus* cultivar *Westar* genome browser (BnPIR, http://cbi.hzau.edu.cn/bnapus), manually corrected the redundant sequence information of the *BnCHYRs*, and named them according to the chromosome position and phylogenetic relationship of BnCHYRs. The genomic sequence length of 24 identified *BnCHYR* genes in *B. napus* showed a wide range from 765 to 3816 bp, indicating large variation. These 24 predicted BnCHYRs encoded polypeptides of 254 to 1271 amino acid residues with molecular weights ranging from 29.82 kDa to 143.78 kDa. Moreover, BnCHYRs isoelectric points (PI) values ranged from 5.43 (BnA01.CHYR) to 7.43 (BnA10.CHYR.1), while their Aliphatic indices ranged from 49.66 to 87.27. The grand average of hydropathy (GRAVY) for all BnCHYRs was predicted to be in the range of −0.636 to −0.121, indicating that these proteins were strong hydrophilicity. Except for six BnCHYR proteins localized in the cytoplasm、nucleus and extracell, five in both the cytoplasm and nucleus, one each in the cytoplasm, in both the cytoplasm and extracell, other 11 proteins were predicted to be located in the nucleus.

**Table 1:** The characteristic of CHYRs identified in *Brassica napus* L

Gene name	Gene Id	Chr	PL (aa)	MW(KDa)	pI	AI	GRAVY	SL
BnA01.CHYR	BnaA01G0241700WE	**A01**	**1247**	**140.06**	**5.43**	**74.35**	**−0.356**	**N**
BnA02.CHYR.1	BnaA02G0075200WE	**A02**	**268**	**31.33**	**6.58**	**55.19**	**−0.550**	**C, N**
BnA02.CHYR.2	BnaA02G0375700WE	**A02**	**305**	**35.14**	**6.09**	**58.10**	**−0.597**	**C, E**
BnA03.CHYR.1	BnaA03G0094800WE	**A03**	**267**	**31.21**	**6.73**	**51.05**	**−0.574**	**C, N**
BnA03.CHYR.2	BnaA03G0111500WE	**A03**	**292**	**33.54**	**6.94**	**49.66**	**−0.636**	**N**
BnA05.CHYR	BnaA05G0326500WE	**A05**	**1244**	**140.19**	**5.69**	**75.47**	**−0.329**	**N**
BnA07.CHYR	BnaA07G0344100WE	**A07**	**1246**	**143.11**	**6.02**	**81.15**	**−0.278**	**N**
BnA09.CHYR.1	BnaA09G0536000WE	**A09**	**293**	**33.28**	**6.15**	**51.30**	**−0.522**	**C, N, E**
BnA09.CHYR.2	BnaA09G0579000WE	**A09**	**1248**	**143.55**	**5.66**	**82.94**	**−0.312**	**N**
BnA10.CHYR.1	BnaA10G0158000WE	**A10**	**295**	**33.85**	**7.43**	**50.95**	**−0.577**	**C, N, E**
BnA10.CHYR.2	BnaA10G0182500WE	**A10**	**267**	**30.96**	**6.41**	**53.97**	**−0.535**	**C, N, E**
BnC01.CHYR	BnaC01G0337900WE	**C01**	**1271**	**143.38**	**5.57**	**73.47**	**−0.367**	**N**
BnC02.CHYR.1	BnaC02G0082600WE	**C02**	**254**	**29.82**	**7.14**	**54.76**	**−0.543**	**C, N**
BnC02.CHYR.2	BnaC02G0491400WE	**C02**	**305**	**35.01**	**6.01**	**58.10**	**−0.582**	**C, N**
BnC03.CHYR.1	BnaC03G0032800WE	**C03**	**267**	**31.12**	**6.92**	**51.80**	**−0.545**	**C, N, E**
BnC03.CHYR.2	BnaC03G0051400WE	**C03**	**292**	**33.53**	**6.94**	**50.99**	**−0.589**	**N**
BnC05.CHYR	BnaC05G0395200WE	**C05**	**1241**	**139.94**	**5.74**	**75.57**	**−0.326**	**N**
BnC06.CHYR.1	BnaC06G0365400WE	**C06**	**975**	**112.61**	**6.82**	**87.27**	**−0.121**	**N**
BnC06.CHYR.2	BnaC06G0427600WE	**C06**	**1247**	**143.78**	**6.05**	**80.57**	**−0.316**	**N**
BnC08.CHYR.1	BnaC08G0365900WE	**C08**	**284**	**32.16**	**6.38**	**51.90**	**−0.461**	**C, N, E**
BnC08.CHYR.2	BnaC08G0413900WE	**C08**	**1248**	**143.74**	**5.81**	**82.55**	**−0.326**	**N**
BnC09.CHYR.1	BnaC09G0417800WE	**C09**	**285**	**32.56**	**6.23**	**63.19**	**−0.420**	**C, N, E**
BnC09.CHYR.2	BnaC09G0452200WE	**C09**	**267**	**31.02**	**6.55**	**53.22**	**−0.532**	**C, N**
Bnscaff406.CHYR	406G0000100WE	**scaffold**	**1013**	**116.57**	**6.46**	**77.12**	**−0.356**	**C**

*Chr* Chromosome, *MW* molecular weight, *PI* isoelectric point, *AI* Aliphatic Index, *SL* subcellular Localization, *PL* Protein Length, *N* Nucleus, *C* Cytoplasm, *C,N* cytoplasm, Nucleus, *C, N,E* cytoplasm, Nucleus, Extracell, *C,E* Cytoplasm, Extracell

**Fig. 1:**
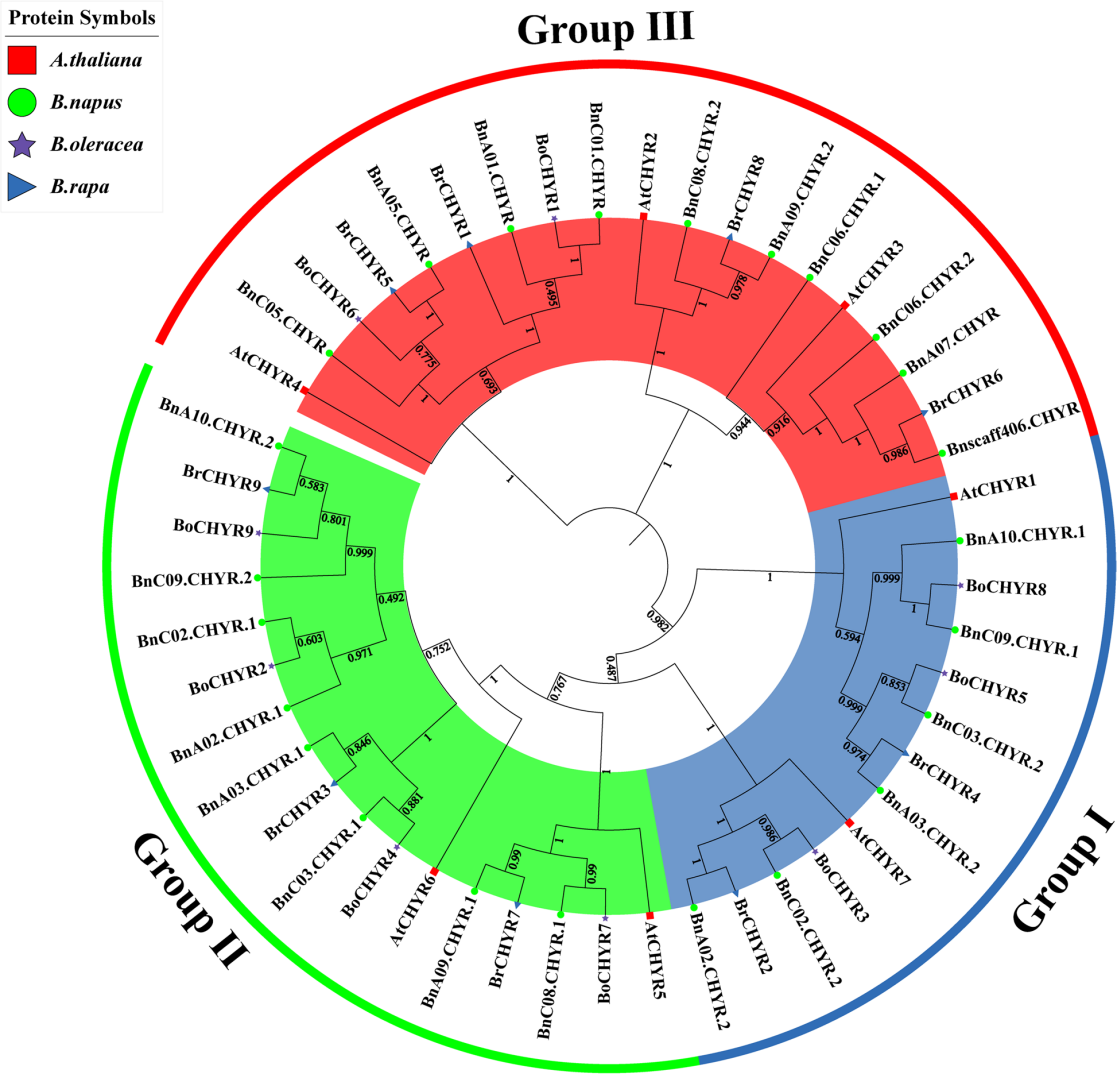
Phylogenetic tree of CHYR proteins from four species in Brassicaceae. Overall, 24 BnCHYRs (Green circle), 9 BoCHYRs (Perple star), 9 BaCHYRs (Blue triangle), and 7 AtCHYRs (Red box) were classified into three groups (Group I-III), and different groups of CHYR proteins were distinguished by different colored backgrounds. The neighbor-joining tree was generated through the MEGA11 program using the amino acid sequences of the CHYRs by the neighbor-joining (NJ) method, with 1000 bootstrap replicates

To elucidate the phylogenetic relationships among the CHYR family, we constructed a phylogenetic tree of 49 CHYR proteins, including 24, 9, 9, and 7 from *B. napus*, *B. rapa, B. oleracea*, and *A. thaliana*, respectively (Fig. 1). Phylogenetic analysis indicated that BnCHYRs could be distinctly divided into three groups (I, II, and III), as reported in soybean, wheat, and *S. alopecuroides* [14, 41, 43]. Our results showed that Group I contained 13 CHYR members (six *BnCHYRs*, two *BraCHYRs*, three *BolCHYRs*, and two *AtCHYRs*), Group II contained 17 CHYR members (eight *BnCHYRs*, three *BraCHYRs*, four *BolCHYRs*, and two *AtCHYRs*), and Group III contained 19 CHYR members (10 *BnCHYRs* (half from the An and Cn sub-genomes), four *BraCHYRs*, two *BolCHYRs*, and three *AtCHYRs*). Notably, nearly half were classified into Group III. Groups within the same sub-family may have similar functions.

### Gene structure and conserved motif composition of BnCHYRs

**Fig. 2:**
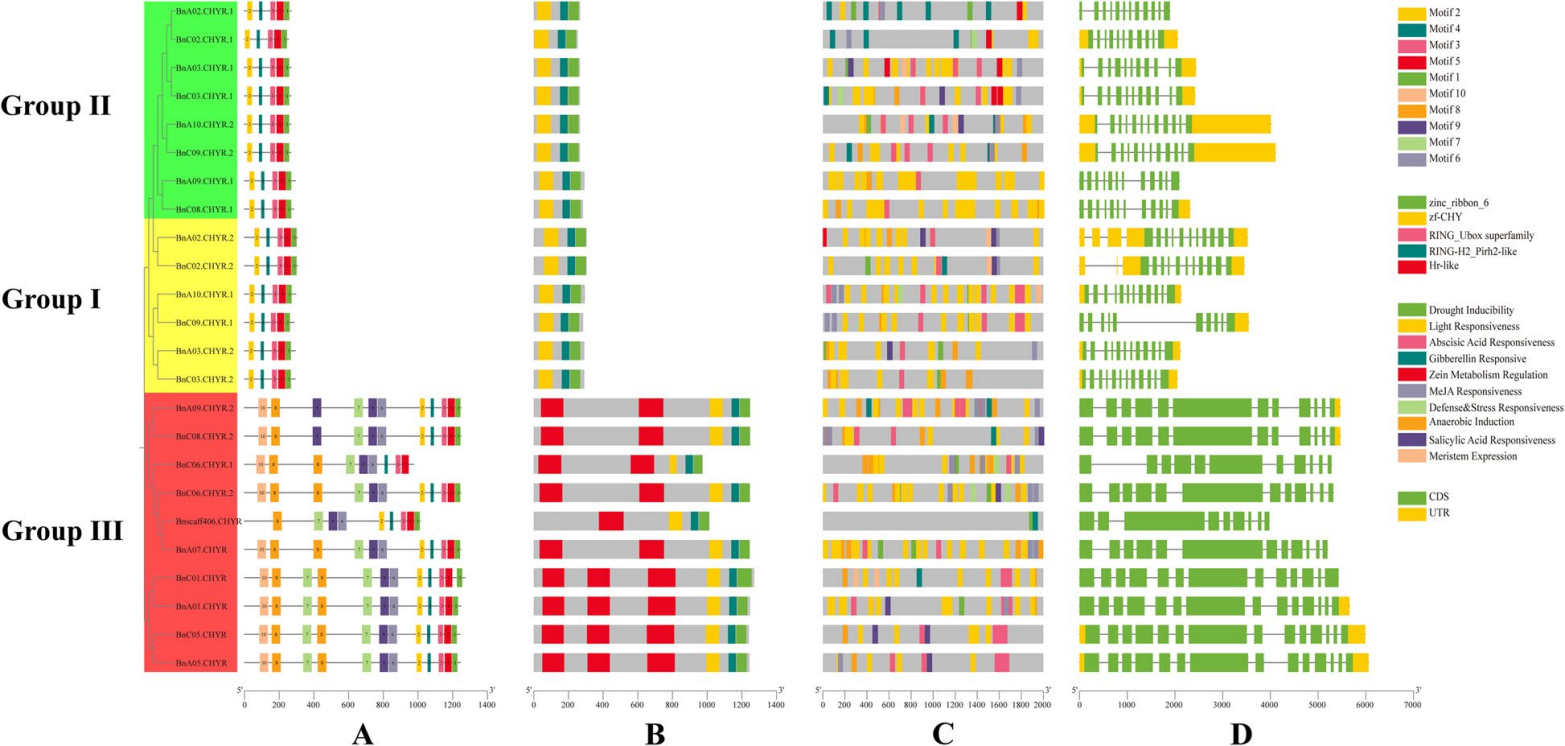
Characterization of CHYR genes in *B. napus*. **A** The conserved motif location of BnCHYRs proteins in *B. napus*. and motif1-motif10 were showed by different colors. **B** The domain location of BnCHYR proteins, CHY-zinc finger, C3H2C3-type RING finger, zinc ribbon and hemerythrin domains (**C**) Distribution of cis-acting elements in the promoters of *BnCHYRs*. Different cis-acting elements are annotated by boxes of different colors. **D** Exon–intron structure features of *BnCHYRs*. Green boxes indicate exons, yellow boxes indicate UTR regions, Blackish-grey lines indicate introns. The bottom scale shows the protein length

To investigate the possible structural evolution of CHYRs in *B. napus*, 24 *BnCHYRs* were analysed for gene structure, conserved motif composition, and *cis* element (Fig. 2). *BnCHYRs* contained 9 to14 exons; the CDS of Group III members was longer than that of the other two groups, and most genes with closer evolutionary relationships had similar exon–intron structures (Table 1; Fig. 2D). The results show that all BnCHYRs contained a CHY-zinc finger, C3H2C3-type RING finger, and zinc ribbon domains (Fig. 2B). These results further confirm the reliability of the identified BnCHYR family members. In addition, Group III members contain one to three hemerythrin domains at the N-terminus, consistent with the results obtained in *A. thaliana*, soybean, and wheat, which may explain the involvement of Group III CHYR members in the regulation of iron homeostasis.

BnCHYRs were subjected to MEME motif analysis, and ten conserved motifs, designated as Motif 1 through 10, were identified in the BnCHYR family (Fig. 2A). Twenty-four BnCHYRs processed only three common motifs, Motif 3, 4, and 5, suggesting that they were the core domains of the CHYR subfamily. In Clade I and II, the BnCHYRs contained five motifs, which were arranged in the same order (Fig. 2A), indicating that these CHYRs may have similar biological functions. Additionally, the hemerythrin domain of Group III members comprises Motif 7, 8, and 10, with conserved Motif 6 and 9 close to this domain (Fig. 2A). These results indicated that conserved motif composition varied among different CHYR subfamilies; however, BnCHYRs with closer evolutionary relationships had more similar conserved domains. BnCHYRs within the same group had similar or identical gene structures and protein motif compositions, strongly supporting the reliability of the group classification.

#### Chromosome distribution and synteny analysis of BnCHYR genes

**Fig. 3:**
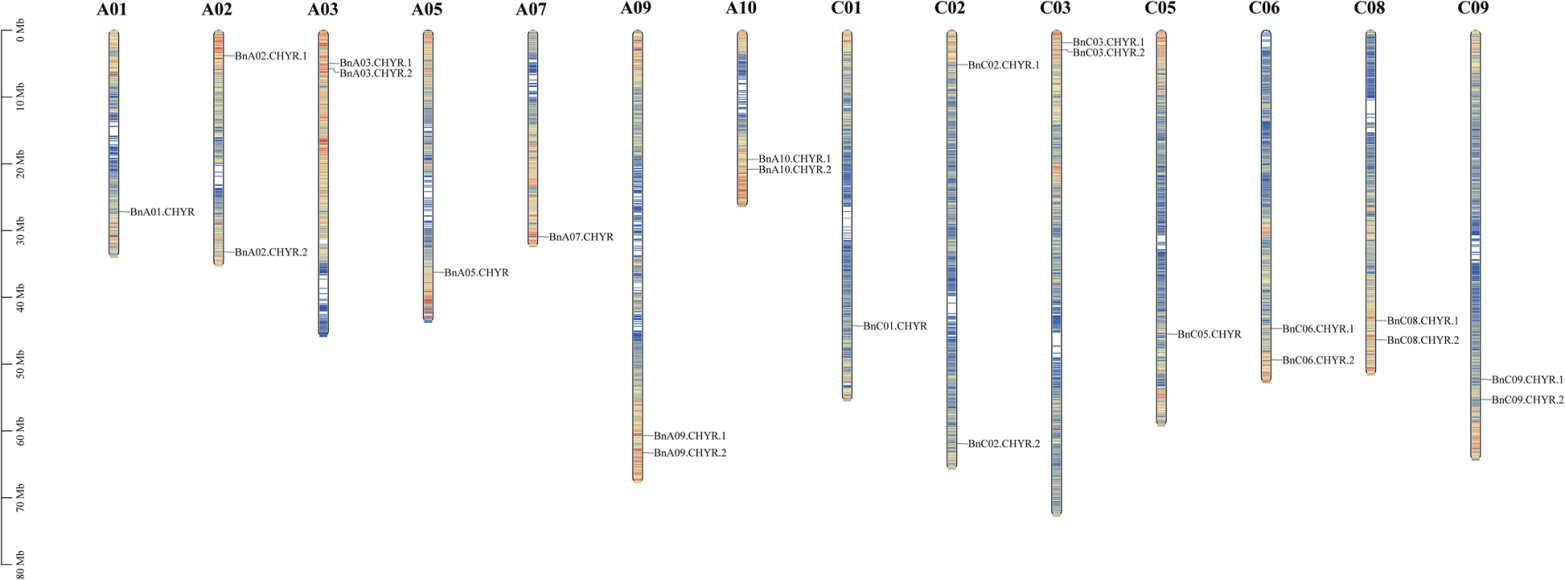
Distribution of *BnCHYRs* on chromosomes of *B. napus*. The name of each chromosome is presented at the top of the corresponding bar, and the gene names are given on the right side. The results on the left indicate the physical position in megabases (Mb)

**Fig. 4:**
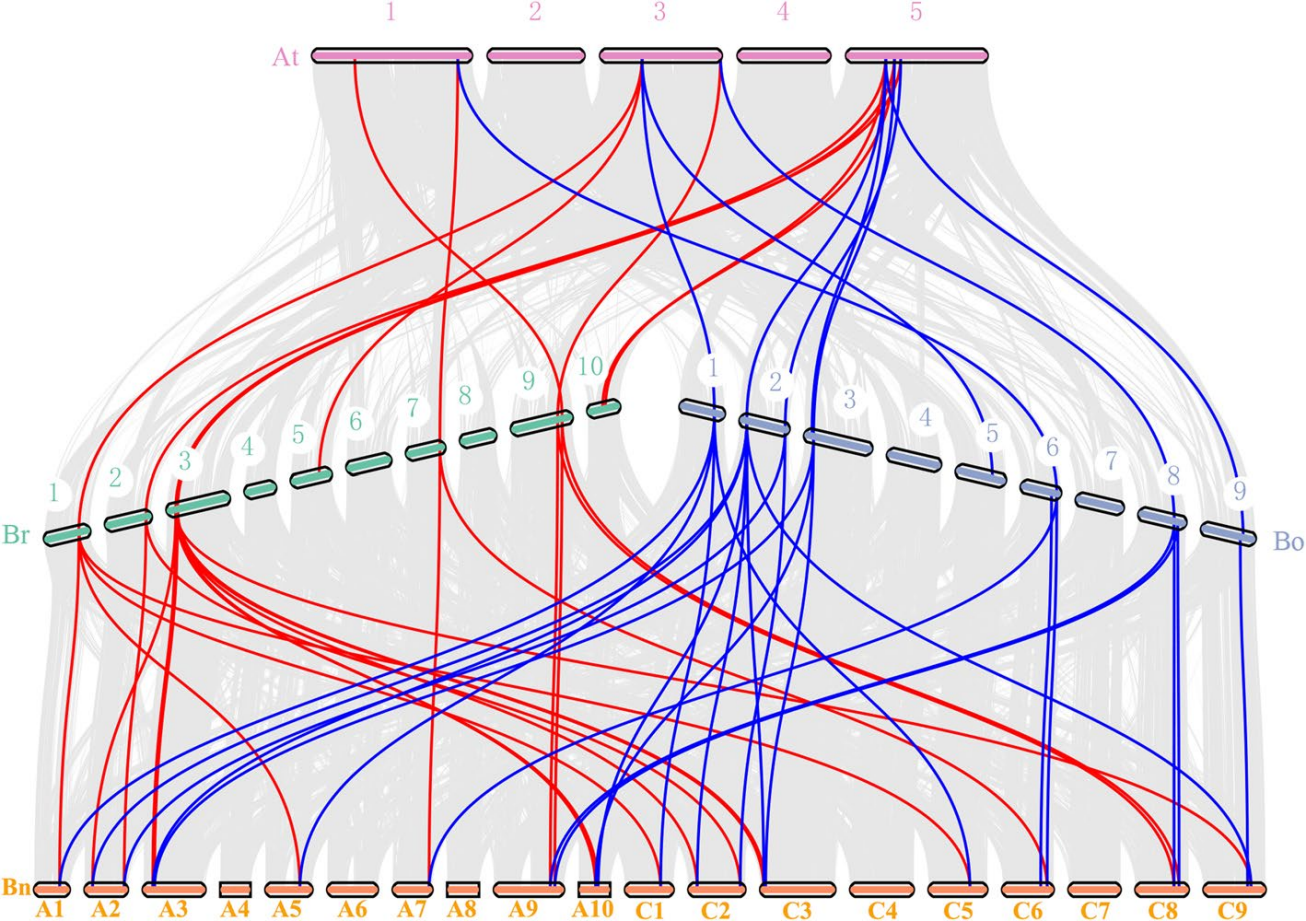
Collinearityy analyses of CHYRs among *B. napus*, *B. rapa*, *B. oleacea*, and *A. thaniana*. The species names with the prefixes “*Bn*”, “*Br*”, “*Bo*”, and “*At*” indicate *B. napus*, *B. rapa*, *B. oleracea* and *A. thaliana*, respectively. The grey lines represent the collinear blocks between different species, while the red lines and blue lines indicates the syntenic *CHYR* gene pairs. The chromosome number is labelled above or behind the each chromosome. The chromosomes of *B. napus* are shown in the form that symbol starting with A represents the chromosome originating from *B. rapa*, and symbol starting with C denotes the chromosome from *B. oleracea*

The chromosomal location and synteny of BnCHYR family members were analzsed based on their genomic sequences (Fig. 3). Briefly, these 24 *BnCHYRs* were unevenly distributed on 14 chromosomes, of which 11 and 12 were in subgenomes A and C, respectively (Table 1; Fig. 3). Notably, *Bnscaff406.CHYR* is located on an unidentified chromosome. Briefly, chromosomes A02, A03, A09, A10, C02, C03, C06, C08, and C09 harboured two *BnCHYRs*, whereas the other chromosomes (A01, A05, and A07) possessed only one gene (Table 1; Fig. 3). Group I members were primarily distributed on chromosomes A02, A03, A09, A10, C02, C03, C09, and C10; Group II on chromosomes A02, A03, A10, C02, C03, and C09; Group III on chromosomes A01, A05, A07, A09, C01, C05, C06, and C08. Our results revealed no significant correlation between *BnCHYR* number and chromosome length. Additionally, four pairs of *BnCHY*R genes from the An subgenome were repeated in tandem on chromosome Bn_A02, Bn_A03, Bn_A09, and Bn_A10, and five pairs of *BnCHYR* genes from the Cn-subgenome were repeated in tandem on chromosomes Bn_C02, Bn_C03, Bn_C06, Bn_C08, and Bn_C09. Genomic analysis of *B. napus* revealed that the An and Cn subgenomes were largely collinear to the corresponding diploid Ar and Co genomes [57, 58]. Most An-Ar and Cn-Co orthologous gene pairs demonstrated similar chromosomal locations (Fig. 4).

*CHYR* gene distribution in *B. rapa* and *B. oleracea* was similar to that of the orthologous *BnCHYR* genes in the *B. napus* An and Cn subgenomes (Fig. 4). Synteny analysis between the An and Cn subgenomes showed high collinearity between Bn_A01-Bn_C01, A02-C02, A03-C03, A04-C04, A05-C05, A06-C06, A07-C07, A08-C08, A09-C09, and A10-C09, and 83.7% of the orthologous gene pairs between *B. rara* and *B. oleracea* were retained as homologous gene pairs between *B. napus* An and Cn chromosomes [57, 58]. Furthermore, 83.3% of *CHYR* gene pairs (10/12 pairs) between *B. rapa* and *B. oleracea* were retained as homologous gene pairs between *B. napus* An and Cn chromosomes.

#### *Cis*-element analysis of BnCHYR genes

The presence of variable *cis*-elements in the promoters of these genes suggests that they perform different functions in plant growth, development, and responses to various stress. *Cis* elements in gene promoter regions regulate the expression of related genes by binding to transcription factors. To further investigate the functions of *BnCHYRs* in plant defence and abiotic stress responses, *cis*-acting element analysis was performed in the 2.0 kb promoter region of *BnCHYRs*. C*is*-acting elements in *BnCHYR* promoter regions were identified using PlantCARE, and the positions of all *cis*-acting elements are marked with boxes of different colours (Fig. 2C). The *cis*-elements were divided into four categories, namely stress-responsive-, light-responsive-, hormone-responsive-, and growth- and development-related-. Ten putative *cis* elements were predicted in the *BnCHYR* promoter. Among them, four were hormone-responsive, Methyl jasmonate (MeJA), gibberellin, and salicylic acid, and the remaining were associated with drought inducibility, light, defense stress, anaerobic induction, and meristem expression. Summary statistics of *cis*-element numbers revealed that “light-responsive elements” were the most abundant, followed by “ABA-responsive elements”, “MeJA-responsive elements”, and “anaerobic induction elements”; “drought inducibility”, and “gibberellin responsiveness” accounted for a considerable number. Only a few elements were present in the remaining four types. As shown in Fig. 2C, most hormone- and stress-responsive elements were specific to certain genes, highlighting their crucial roles in hormone and stress response mechanisms.

Notably all *BnCHYRs* contained *cis*-acting elements associated with hormone regulation, with most being linked to stress or hormonal regulation. The *BnCHYR* promoter included several stress and hormone response elements, particularly stress response elements, including the anaerobic response element (ARE) and MBS (MYB binding site) (Fig. 2C). These results are similar to those predicted based on the CCREs of the CHYR family gene promoters in soybean, wheat, and *S. alopecuroides* [14, 42, 43]. Additionally, Group I members have abundant ARE. The G-box in *BnCHYR* promoters can interact with bZIP or bHLH transcription factors to participate in biological processes [59]. These results indicate that *BnCHYRs* play significant roles in plant growth, development, and response to various stresses in *B. napus*. Furthermore, some *cis* elements were responsive to abiotic stresses, including drought, defence, stress, and anaerobic responses. A large difference was observed in the type and number of *cis* elements among the different groups or even within the same group.

#### Expression patterns of BnCHYRs under abiotic stress

**Fig. 5:**
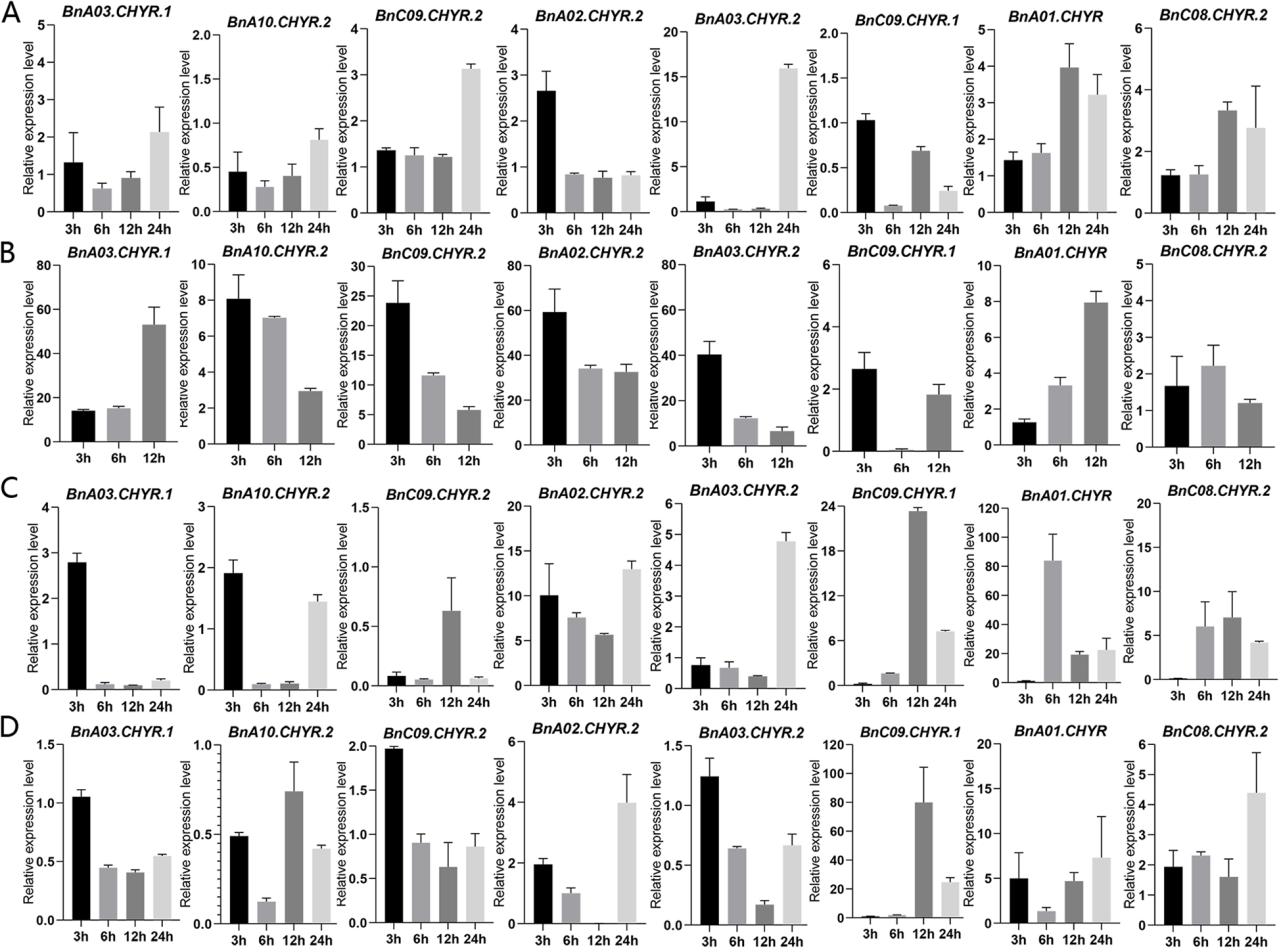
Expression patterns of *BnCHYR* genes in response to cold (**A**), heat (**B**), salt (**C**) and PEG (polyethylene glycol) (**D**) treatments determined by real-time PCR. Rapeseed seedlings were sampled after 3, 6, 12 and 24 h under stress conditions. The expression level of the rapeseed *Bnactin7* gene was used as the internal control to standardize the RNA samples for each reaction. The values were the mean ± SE from three samples

Based on the *cis*-element analysis, these *BnCHYRs* were predicted to be involved in the response to abiotic stress. To further confirm this hypothesis, the expression levels of eight representative *BnCHYRs* from three groups under drought, salt, cold, and heat stresses were validated using qRT-PCR; the analysed genes included *BnA02.CHYR.2*, *BnA03.CHYR.2*, and *BnC09.CHYR.1* (Group I); *BnA03.CHYR.1*, *BnC09.CHYR.2*, and *BnA10.CHYR.2* (Group II); *BnA01.CHYR* and *BnC08.CHYR.2* (Group III) (Fig. 5; Fig S2). We conducted gene expression analyses of *BnCHYRs* in rapeseed under salt, drought, cold, and heat stresses at 3, 6, 12, and 24 h using the 0 h treatment as the control. The eight *BnCHYRs* were significantly induced or repressed by multiple treatments, which is consistent with their functional predictions based on *cis* element analysis. None of the eight genes showed consistent expression patterns under the four treatments (Fig. 5).

*BnCHYRs* expression was induced by heat stress, showing different expression patterns (Fig. 5B). *BnA02.CHYR.2* and *BnA03.CHYR.2* were significantly up-regulated by more than 59-fold and 40-fold, respectively, under heat stress compared with controls (Fig. 5B). *BnCHYRs* exhibited differential expression patterns in seedlings in response to salt, cold, and drought stress. NaCl treatment induced the expression of these *BnCHYR* genes, except for *BnC09.CHYR.2* (Fig. 5C). After cold stress treatment, most of these genes were upregulated, while *BnA10.CHYR.2* and *BnC09.CHYR.1* expression levels were suppressed compared with controls (Fig. 5A). Under drought stress, *BnC09.CHYR.2*, *BnA02.CHYR.2*, *BnC09.CHYR.1*, *BnA01.CHYR*, and *BnC08.CHYR.2* were up-regulated, and *BnA03.CHYR.1*, *BnA10.CHYR.2*, and *BnA03.CHYR.2* expression levels were suppressed compared with controls (Fig. 5D). These results suggest that *BnCHYRs* respond differently to different stresses and play various regulatory roles in abiotic stress resistance.

#### Functional analysis of *BnA03.CHYR.1*

**Fig. 6:**
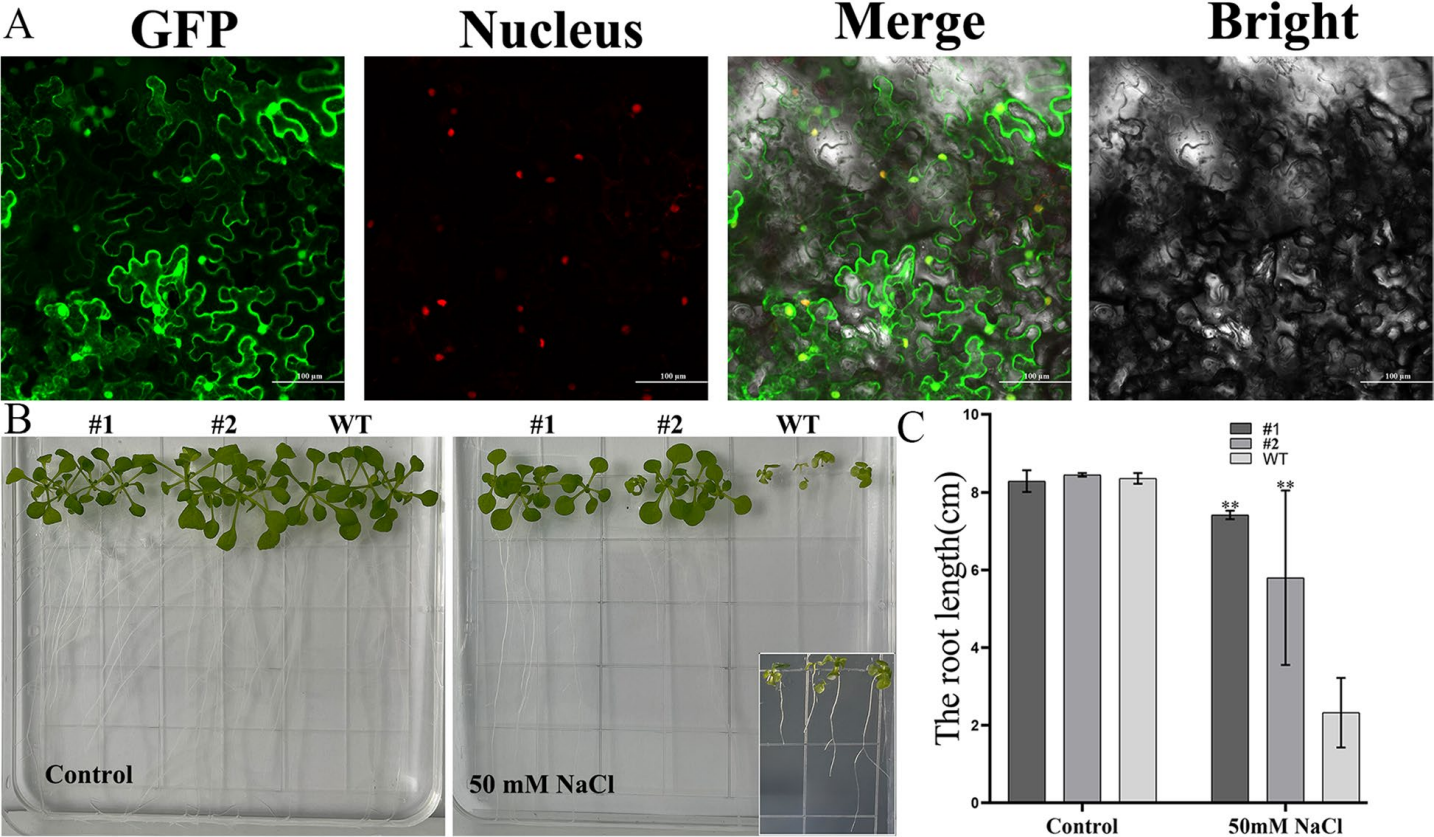
Subcellular localization analysis and salt tolerance of transgenic *A. thaliana* plants. **A** Subcellular localization analysis of BnA03CHYR.1 protein. **B** The growth of WT and OEs plants under 1/2MS (control) and 1/2MS + 50 mmol/L NaCl. Insert show magnification of the wild-type (WT) seedlings cultivated on a medium supplemented with 50 mM NaCl. **C** The primary root length of WT and OEs seedlings on 1/2MS with 0 and 50 mmol/l NaCl. Values are means ± SDs (*n* = 3). ** indicates significant difference between OES and WT by one-way ANOVA with Student’s t-test (*,* P* < 0.05; **, *P* < 0.01)

In addition, the subcellular localisation of BnA03.CHYR.1 was monitored through fusion with GFP protein. Vectors expressing BnA03.CHYR.1::GFP alone were transformed into tobacco cells using the transient expression method, and fluorescence was observed using confocal microscopy (Fig. 6A). The BnA03.CHYR.1::GFP fluorescence signal was localised in the cytoplasm and nucleus (Fig. 6A). To investigate the biological function of *BnA03.CHYR.1* under salt stress, we ectopically overexpressed this gene in *A. thaliana*. Three OEs with high expression levels were selected for functional analysis of *BnA03.CHYR.1* under salt stress response. OE seedlings were similar to those of the WT on 1/2MS medium without NaCl. However, OE seedlings were all significantly stronger than those of the WT on 1/2MS medium containing 50 mmol/L NaCl (Fig. 6B). Primary root growth is a crucial indicator of plant tolerance to salt stress; hence, the root lengths of the WT and OE plants were measured. OE and WT root lengths were approximately 8.5 cm on 1/2MS medium without NaCl; however, WT plants had significantly shorter roots than OE plants when grown on 1/2MS supplemented with 50 mmol/L NaCl (Fig. 6C). Based on the above results, it could be concluded that *BnA03.CHYR.1* may be a positive regulator in response to salt stress.

## Discussion

As a member of the RING-type E3 ubiquitin protein ligase family, *CHYR* plays a vital role in regulating plant growth, development, and responses to abiotic and biotic stresses [15, 20, 26]. CHYR proteins have been identified in many plant species including maize [41], rice [30], Populus [28], *A. thaliana* [20], soybean [14], wheat [42], tomatoes [29], and *Sophora alopecuroides* [43]. To explore the genetic information of the *CHYR* gene family in *B. napus* and their roles in plant responses to abiotic stress, we analysed the entire *B. napus* genome. In the present study, 24 *CHYR* genes were identified in *B. napus*, and classified into three groups, consistent with the identification of CHYR in other species. All BnCHYRs contained the CHY-zinc finger, C3H2C3-type RING finger and zinc ribbon domains. Only Group III members contained one to three additional hemerythrin domains at the N-terminus, which may play vital roles in regulating iron homeostasis [23]. The CHYR protein family was conserved to some extent among species, suggesting that they play crucial roles in plant growth, development, and adaptation to adverse environmental conditions.

Additionally, CHYR gene collinearity was analysed in Brassica, using three representative species as examples. Almost all CHYRs in the *B. napus* An and Cn sub-genomes had syntenic genes in the diploid Ar and Co sub-genomes, respectively. This was expected given the relatively recent formation of *B. napus* from its progenitors [57]. Most gene pairs between the same sub-genomes (An, Ar, Cn, and Co) had similar chromosomal locations. Several gene pairs are located in homologous chromosome segments from different sub-genomes, which may result from homologous non-reciprocal translocations [60] or homologous exchanges [57, 61].

The CHYR protein, a RING-type E3 ubiquitin ligase, participates in the regulation of plant growth, development, and environmental adaptation through ubiquitination. The expression of 14 of the 18 *CHYR* genes identified in *S. alopecuroides* was significantly triggered by salt, alkaline, and drought stress [43]. The expression of all TaCHYRs identified in wheat was induced by drought stress, showing similar expression patterns [42]. *TaCHYR2* and *TaCHYR4* were evident upregulated under salt stress, reaching their highest expression levels at 36 h, whereas *TaCHYR8* and *TaCHYR9* were significantly down-regulated [42]. *GmCHYR15* expression was repressed by dehydration, salt, and alkaline stresses, whereas expression of *GmCHYR3*and *GmCHYR5* were induced by these stresses [14]. In the present study, expression levels of six of the eight *BnCHYRs* identified in *B. napus* were induced by salt, cold, and drought stresses. Expression of the eight *BnCHYRs* was significantly triggered by heat stress, showing different expression patterns. These findings reveal that *BnCHYRs* play crucial roles in *B. napus* in response to abiotic stress.

RING- type E3 reportedly plays critical roles in regulating plant responses to abiotic stress and ABA signalling [62–69]. Recent studies have shown that several E3 ligases regulate these responses by targeting and mediating the degradation of salt stress-related proteins. *AtAIRP3*/LOG2, *AtPp2-B11*, *AtSDIR1*, and *AtSTRF1* act as positive regulators of salt tolerance [70–75], whereas *AtPUB30*, *AtPPRT1*, and *AtXBAT35.2* negatively regulate the response to salinity stress [68, 76, 77]. *OsRHP1*, *OsSIRF1*, *OsSIRP2*, and *OsSIRH2-14* are positive regulators of salt tolerance [78–81], while *OsDSG1*, *OsMAR1*, *OsSIRP1*, *OsSIRP3*, *OsSIRP4*, *OsSRFP1*, *OsSADR1*, *OsDIRP1*, *OsDHSRP1*, and *OsMSRFP* negatively regulate the response to salinity stress [82–91]. In wheat, PUB1, ZNF, and TaSDIR1 act as positive regulators of salt tolerance [92–94], whereas *AtPUB15* and *PUB26* negatively regulate the response to salinity stress [95, 96]. MfSTMIR is a specific ERAD E3 ligase, that may participate in ERAD through its interaction with MtUBC32 and MtSec61γ to relieve the ER burden under salt stress [97]. The RING finger E3 ligase SpRing positively regulates salt stress signalling in salt-tolerant wild tomato species [98]. In cotton, GhSARP1 negatively regulates salt stress tolerance, and its overexpression enhances salt stress sensitivity [99]. The apple RING finger E3 ubiquitin ligase MdMIEL1 negatively regulates salt and oxidative stress tolerance [100]. In *B. napus*, *BnA03.CHYR.1*, a RING finger E3 ligase, was induced under.high-salinity stress conditions, and its overexpression in *A. thaliana* improved salt stress tolerance (Figs. 5C and 6B). However, exact mechanism by which *BnA03.CHYR.1* affects salt stress tolerance remains unclear.

Studies have shown that RING E3 ligases play crucial roles in responses to abiotic stress via different mechanisms, such as signal transduction, hormone sensing and transcription factors [80, 101–104]. Ring-type E3 ubiquitin ligases contain a RING zinc-finger domain, that plays a critical role in abiotic stress responses via the ABA signalling pathway. TaCHYRs regulate plant adaptive responses to abiotic stress via ABA-mediated signalling, particularly by modulating the stability of bZIP and bHLH transcription factors [42, 59, 105]. RING-type E3 ubiquitin ligases of the CHYR regulate enzyme activity (via ubiquitination), and the expression of transcription factors (e.g., WRKY and MYB) to regulate stress responses in plants [27, 33, 100, 106, 107]. In mammals, the RING finger E3 ligase XIAP [108] targets over 50 substrates across various cellular components, regulating diverse biological function. Studies across a diverse plant group have revealed that E3 ligases are crucial in regulating pathways under high salt condition. Our results show that *BnA03.CHYR.1* overexpression in *A. thaliana* significantly improved salt tolerance. However, the underlying mechanism by which it regulates the salt response in rapeseed remains unknown. Therefore, further investigations are required to uncover how *BnA03.CHYR.1* is functionally correlated with the activation of defence mechanism against salt conditions.

## Conclusions

In the current study, we identified and characterized the CHYR family in B. napus, including analyses of phylogeny, cis-elements, gene structure, conserved motifs, chromosome localization, and response to abiotic stress. Notably, the overexpression of *BnA03.CHYR.1* in *A. thaliana* suggested the positive role of *BnA03.CHYR.1* in regulating salt tolerance. Therefore, *BnA03.CHYR.1* will be conducive to in-depth exploration of the molecular mechanisms of salt tolerance. These results provide a basis for further analysis of *BnCHYR* genes to determine their function and elucidate the molecular mechanisms underlying the response of *B. napus* to abiotic stress.

## Supplementary Information

Supplementary Material 1.

Supplementary Material 2.

## Data Availability

Data is provided within the manuscript or supplementary information files.
