# Supplementary figures and images for: Genome-wide analysis of CHYR gene family and BnA03.CHYR.1 functional verification under salt stress in Brassica napus L
Source: BMC Plant Biol. 2025 Mar 20;25:363. doi: 10.1186/s12870-025-06343-x (PMC11924726; doi:10.1186/s12870-025-06343-x)

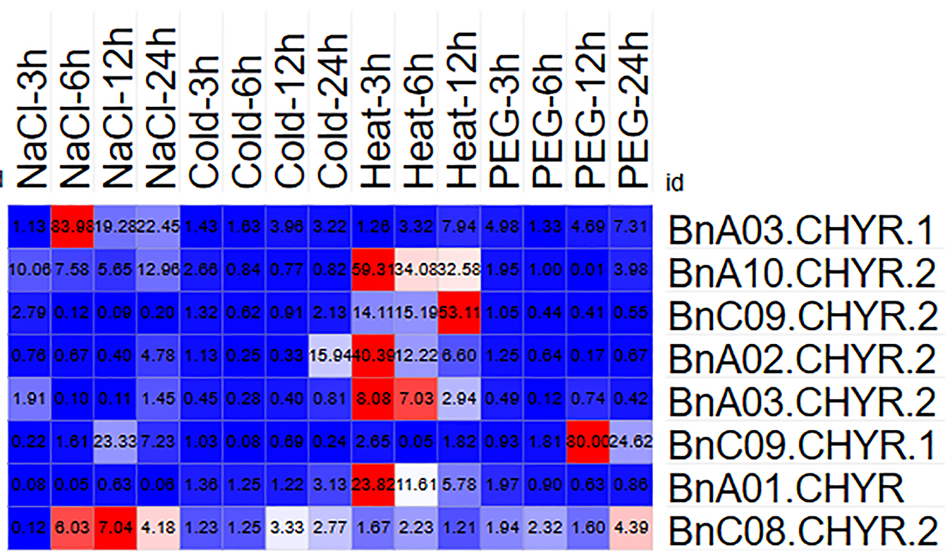

Supplement: Supplementary file 2 — Supplementary Material 2. [file 12870_2025_6343_MOESM2_ESM.tif]
